# Celia’s Encephalopathy (*BSCL2*-Gene-Related): Current Understanding

**DOI:** 10.3390/jcm10071435

**Published:** 2021-04-01

**Authors:** Sofía Sánchez-Iglesias, Antía Fernández-Pombo, Silvia Cobelo-Gómez, Álvaro Hermida-Ameijeiras, Helena Alarcón-Martínez, Rosario Domingo-Jiménez, Alejandro Iván Ruíz Riquelme, Jesús R. Requena, David Araújo-Vilar

**Affiliations:** 1 UETeM-Molecular Pathology Group, Department of Psychiatry, Radiology, Public Health, Nursing and Medicine, IDIS-CIMUS, University of Santiago de Compostela, 15782 Santiago de Compostela, Spain; sofia.sanchez@usc.es (S.S.-I.); antiafpombo@gmail.com (A.F.-P.); silviacobelog@gmail.com (S.C.-G.); alvaro.hermida@usc.es (Á.H.-A.); 2 Division of Endocrinology and Nutrition, University Clinical Hospital of Santiago de Compostela, 15706 Santiago de Compostela, Spain; 3 Division of Internal Medicine, University Clinical Hospital of Santiago de Compostela, 15706 Santiago de Compostela, Spain; 4 Department of Pediatric Neurology, Hospital Virgen de la Arrixaca and IMIB-Arrixaca, CIBERER-ISCIII, 28029 Madrid, Spain; helenaalarconmartinez@yahoo.com (H.A.-M.); mrosario.domingo@gmail.com (R.D.-J.); 5 German Center for Neurodegenerative Diseases (DZNE), 72076 Tübingen, Germany; alejandro-ivan.ruiz-riquelme@dzne.de; 6 Prion Laboratory, CIMUS Biomedical Research Institute, University of Santiago de Compostela-IDIS, Santiago de Compostela, 15782 Santiago de Compostela, Spain; jesus.requena@usc.es

**Keywords:** Celia’s encephalopathy, PELD, seipin, *BSCL2*, congenital generalized lipodystrophy, neurodegeneration

## Abstract

Seipin, encoded by the *BSCL2* gene, is a protein that in humans is expressed mainly in the central nervous system. Uniquely, certain variants in *BSCL2* can cause both generalized congenital lipodystrophy type 2, upper and/or lower motor neuron diseases, or progressive encephalopathy, with a poor prognosis during childhood. The latter, Celia’s encephalopathy, which may or may not be associated with generalized lipodystrophy, is caused by the c.985C >T variant. This cytosine to thymine transition creates a cryptic splicing zone that leads to intronization of exon 7, resulting in an aberrant form of seipin, Celia seipin. It has been proposed that the accumulation of this protein, both in the endoplasmic reticulum and in the nucleus of neurons, might be the pathogenetic mechanism of this neurodegenerative condition. In recent years, other variants in *BSCL2* associated with generalized lipodystrophy and progressive epileptic encephalopathy have been reported. Interestingly, most of these variants could also lead to the loss of exon 7. In this review, we analyzed the molecular bases of Celia’s encephalopathy and its pathogenic mechanisms, the clinical features of the different variants, and a therapeutic approach in order to slow down the progression of this fatal neurological disorder.

## 1. Introduction

Celia’s encephalopathy or progressive encephalopathy with or without lipodystrophy (PELD), MIM: # 615924, is a pediatric neurodegenerative condition with a fatal prognosis, usually during the first decade of life. This disorder, described by our group in 2013 [1], is extremely rare and is generally caused by the c.985C >T variant of the *BSCL2* gene in homozygosity or compound heterozygosity. PELD could be considered as a particular variant of congenital generalized lipodystrophy type 2 (CGL2) [2] with a devastating neurological involvement.

In this review, we intended to make a detailed description of the natural history of this disease, its associated complications, and the different variants that have been reported in recent years, both from a molecular point of view and in its clinical manifestations. We also addressed the molecular bases that explain the neurological picture and a possible therapeutic approach.

## 2. *BSCL2* Gene

This gene is located on the long arm of chromosome 11, at position 11q13, with up to 27 transcripts having been reported. The *BSCL2* gene principally encodes three seipin isoforms under natural conditions, according to Ensembl Genome Browser (https://www.ensembl.org/Homo_sapiens/Gene/Summary?db=core;g=ENSG00000168000;r=11:62690275-62709845) (accessed 30 March 2021), 462 (*BSCL2*-203 ENST00000360796.9; CCDS44627), 398 (*BSCL2*-205/207/210, ENST00000403550.5; ENST00000407022.7; ENST00000421906.5; CCDS8031), and 287 (*BSCL2*-201, ENST00000278893.11; CCDS55769) amino acids long. *BSCL2*-205/207/210 was the first transcript to be described [2], and *BSCL2*-203 is the same as *BSCL2*-210/207/205 except for an N-terminal extension of 64 amino acids encoded by exon 1 and part of exon 2. The protein translated from the short transcript is different from the other transcripts, as exon 7 is skipped in *BSCL2*-201 and the reading frame is different from exon 6 to exon 10 [1].

For years, it has been known that the *BSCL2* gene is expressed in humans mainly in the central nervous system (CNS), pituitary, and testes [2]. More recently, our group [3] has found differences in the expression of different *BSCL2* transcripts. Thus, the *BSCL2*-203 transcript is expressed mainly in the CNS (80%), while in other tissues the expression of the *BSCL2*-203 and *BSCL2*-205/207/210 transcripts is similar (50:50). Finally, the transcript that encodes the shorter seipin has a negligible expression (<1%), not only in CNS but also in non-nervous tissues [3].

## 3. Seipin Protein

### 3.1. Structure and Location

Seipin is a resident protein of the endoplasmic reticulum (ER) that consists of two cytoplasmic domains, N- and C-terminal domains, two transmembrane domains, and a luminal loop [4].

The longest isoform differs from the intermediate isoform in that it has an additional 64 amino acids in the N-terminal cytoplasmic domain. The short isoform lacks the second transmembrane domain, so that from amino acid 111, referring to transcript *BSCL2*-203, all the protein is found within the lumen of the ER. Single particle electron microscopy revealed that this protein forms donut-shaped homooligomers with a central cavity [5]. By means of cryo-electron microscopy, it was found that this ring or toroid consists of 11 units in humans [6] and 12 in flies [7]. Using high-resolution atomic models of the luminal domains, the presence of hydrophobic α helices was verified at the inner edge of the oligomeric ring, which probably bind to the ER membrane [8]. Furthermore, the luminal domain contains a β sandwich that resembles the lipid-binding protein Niemann Pick C2 (NPC2) [6,7,8].

### 3.2. Role in Adipogenesis

Seipin functions at the interface of the lipid droplets (LD) and ER, crucial for the early formation of normal LD, but is also involved in maintenance of mature LD [8]. On the other hand, it has also been attributed a role in modulating the synthesis of triglycerides and phospholipids [9,10,11], and in the transmission of nerve impulses in neurons [12]. It is very likely that their functions are different depending on the tissue, and thus seipin is involved in lipid homoeostasis by restricting lipogenesis and LD accumulation in non-adipocytes, while promoting adipogenesis to accommodate excess energy storage [13].

How seipin influences the formation of LD is still not well understood. LD are organelles surrounded by a phospholipid monolayer that encloses a nucleus filled with neutral lipids, most commonly triacylglycerol (TAG) and sterol esters. LD play a crucial role in cellular metabolism and nutrient availability, in such a way that lipids stored during conditions of excess nutrients are mobilized for the production of energy during starvation or for the synthesis of phospholipids that are critical in membrane formation. There is a growing body of evidence implicating LD in many cellular processes, including ER stress response, protein degradation, membrane trafficking and signal transduction, virus assembly, and even for temporary storage of proteins [14]. LD also buffer an excess of potentially toxic lipids, playing a prominent role in the prevention of lipotoxicity and oxidative stress [15].

The first clues about the involvement of seipin in LD biogenesis come from studies in yeast. In knockout models of Sei1p, the yeast seipin homologue, an alteration in the formation of LD has been observed, with very small and giant LD. These studies conclude that seipin has different functions, both in promoting early onset of LD and in regulating LD morphology [16,17,18].

Apparently, the seipin toroid would be floating rapidly in the ER membrane [19], able to detect small accumulations of neutral lipids that are found forming lenses within the ER phospholipid bilayer [19]. The expansion of the neutral lipid lens results in the LD sprouting from the ER membrane [19,20]. This budding requires the participation of coat proteins that induce membrane curvature, such as COPII, although the mechanisms that determine that LD emerge towards the cytosol and not towards the ER lumen seem related to the phospholipid composition of the ER membrane and surface tension [15]. Thus, surface tension seems to be particularly important for acquiring the rounded shape of LD, while the phospholipid composition affects budding efficiency mainly through geometric effects: Conical-shaped molecules, such as diacylglycerol or phosphatidylethanolamine do not promote budding, whereas molecules with opposite geometry, such as lysophospholipids, do [21].

Furthermore, the membrane tension also seems to condition the directionality of the budding process [15]. On the other hand, phospholipids and/or proteins could mask the oil–water interface, affecting the surface tension. Therefore, the differential composition of proteins and/or lipids between the membrane monolayers is sufficient to induce stress imbalances that ultimately determine the directionality of budding. Although in some cases LD have been detected in the ER lumen, in most cases budding of the LD occurs almost exclusively towards the cytosol, suggesting that the stress on lipid monolayers of the membrane of the ER is strictly controlled [15].

In particular, seipin in its toroidal formation would anchor the nascent LD around the bridge between ER and LD [22]. This, in association with other partners, favors the flow of lipids into the LD, thus promoting their growth, until they are separated from the ER in the cytoplasm [23]. However, the mechanism by which the latter occurs has not been elucidated to date.

Recently, other proteins have been discovered that, associated with seipin, are crucial in the formation of LD. One of them is Promethin or lipid droplet assembly factor 1 (LDAF1) [24]. It appears that seipin and Promethin/LDAF1 form a complex, collaborating functionally to the growing LD [25]. In more recent studies [26], it has been suggested that Promethin/LDAF1 could be placed in the center of the seipin toroid, and that the hydrophobic helical domain of LDAF1 could adopt a double hairpin topology, which would allow the protein to anchor on the ER membrane or on the LD surface. Hypothetically, the seipin-LDAF1 complex would contain a large number of hydrophobic domains in its core, which could catalyze the nucleation of the lipid lens by providing a space devoid of phospholipids that normally interfere with triacylglycerides nucleation. As an alternative hypothesis, LDAF1 could form a channel within the seipin toroid, facilitating lipid transfer to growing LD in collaboration with the putative NPC2-like lipid-binding domain of seipin [26]. Seipin could also trap the TAGs through its luminal hydrophobic helices, acting as a catalyst to group the TAGs from the monomers dissolved within the seipin ring, thus generating a binding interface favorable to LDAF1 [27]. In any case, it appears that the accumulation of TAG could break the seipin-LDAF1 interaction, allowing LDAF1 to cover the LD [8]. Interestingly, the fact that the loss of seipin is associated with the disappearance of LDAF1 could suggest that the pathologies associated with the loss of seipin function are related to the absence of LDAF1 [8].

Another protein that seems to collaborate with seipin in the formation of LD, at least in yeast, is PEX30 [28]. PEX30 is an ER resident protein with well-established roles in peroxisome formation [29], for which there is evidence that it also participates in the initial formation of LD in collaboration with seipin [28]. Thus, PEX30 and seipin are enriched in ER domains where both LD and pre-peroxisomal vesicles appear to form, and the simultaneous deletion of seipin and PEX30 inhibits the budding of LD, resulting in the accumulation of neutral lipids within the ER membrane, with an abnormal composition [15]. Apparently, the seipin-PEX30 association could control the lipid composition at the LD budding sites, probably through the regulation of certain lipid-modifying enzymes, such as glycerol-3-phosphate acyltransferases (GPAT) [10].

LD are also found within the nucleus, although their biogenesis and functions are less well understood. It has been proposed that nuclear LD production may be an important part of cellular stress resistance [30]. In yeasts, Romanauska et al. [31] demonstrated that seipin is found in the inner nuclear membrane (INM) and that it is involved in the formation of LD. However, in mammalian cells, seipin was not found in this location [32], although it was detected in the proximity of the outer nuclear membrane, which is part of the ER, so that the formation of LD in the INM is independent of the seipin. However, it does seem that seipin indirectly influences the biogenesis of LD in these cells through its effect on the expression of lipin-1 and the intracellular distribution of phosphatidic acid (PA) [32,33]. The explanation for these differences between yeast and mammalian cells is yet to be discovered. This aspect is relevant because, as will be seen later, our group found seipin aggregates inside the nuclei of hypothalamic neurons from a patient with PELD [34].

Other functions of seipin in the formation of LD have been postulated, generally related to association with other proteins that would act as partners. Thus, for example, seipin is also believed to be involved in adipogenesis, although its exact role is yet to be determined. It is known that the loss of seipin leads to defective adipogenesis [35,36]. This may be due to changes in PPAR**γ** signaling in response to alterations in phospholipid metabolism. Seipin negatively regulates the activity of glycerol-3-phosphate acyltransferase (GPAT). Therefore, loss of functional seipin would result in high levels of PA, which could inhibit PPAR**γ** signaling [10], a key transcription factor in the initiation of adipogenesis. Seipin has also been reported to spatially coordinate two lipid metabolism enzymes, lipin phosphatidic acid phosphatase and acylglycerol phosphate acyltransferase (AGPAT), and the uncoupling of these two enzyme activities can also lead to a local increase in PA [9]. Strikingly, the NPC2-like seipin domain has been found to bind PA and other anionic phospholipids in vitro, pointing to a possible direct role in lipid manipulation [6]. Other alternative links of seipin with adipogenesis would be the remodeling of the actin cytoskeleton [37] or an effect on calcium homeostasis in the ER [38].

In addition to ER, LD interacts with other cellular organelles such as mitochondria and peroxisomes. Peroxisomes are key metabolic organelles that contribute to the metabolism of cellular lipids, e.g., the β-oxidation of very long-chain fatty acids and the synthesis of myelin sheath lipids, as well as the metabolism of reactive oxygen species (ROS), in particular hydrogen peroxide. In addition, in recent years, it has become clear that peroxisomes also serve crucial non-metabolic functions, e.g., in responses to cellular stress, the fight against pathogens and antiviral defense, as cell signaling platforms and in healthy aging. These findings indicate that peroxisomes are also “protective” organelles with a broader meaning in human health and a potential impact on a host of globally important human diseases such as neurodegeneration, obesity, cancer, and age-related disorders [39,40].

### 3.3. Role in Central Nervous System

That seipin must have a relevant role in the functioning of the nervous system seems quite obvious in light of what we already know in relation to the formation of LD, its predominant expression in the CNS and the neurological diseases to which it is associated. That said, we are still far from understanding what its real role is in nervous tissues.

On the one hand, the brain is the second most lipid-rich organ, and stores 20% of the body’s total cholesterol [41,42]. On the other, it is known that the alteration in the lipid composition of CNS cells affects cell function and normal neuronal activity [43,44], and that lipid homeostasis is necessary to maintain neuronal function and synaptic plasticity [45], with lipid dysregulation being related to neurodegenerative disorders, including Alzheimer’s and Parkinson’s disease [46,47,48]. While LD appear to be mainly located in the microglia [49], there is evidence that they are formed in all brain cells [48]. Although the subventricular zone is the most studied brain region with respect to LD [50] formation, other structures, such as the frontal cortex, hippocampus, olfactory bulbs, and hypothalamus, have been shown to accumulate LD [48]. There is evidence relating the formation of LD with neurodegenerative diseases. Thus, it has been shown that the number and size of LD in the brain increase as rats age [51], that the LD content in flies’ brains is correlated with accelerated neurodegeneration [52], that toxic fatty acids from neurons are transferred to astrocyte LD through ApoE-positive lipid particles [53], and that a subset of lipid-accumulating microglia has its function altered with age [49]. Therefore, aging, inflammation, and oxidative stress, all of which are linked to the pathogenesis of neurodegenerative diseases, have been related to the formation of LD [49,51,54,55].

Regarding the specific functions of seipin in the CNS, most of the studies have been carried out in rodents, with many uncertainties still remaining. Thus, although in rodents seipin is expressed mainly in the basal forebrain, hippocampus, hypothalamus, and dorsal and ventral brainstem, with the expression in the cortex lower [56,57], the findings in humans observed by our group are different, as we have found the expression of *BSCL2* to be higher in the caudate and putamen nuclei, cerebellum, and pons, and mainly in the forebrain and rhombencephalon [3]. This finding has special relevance in light of what was observed in the imaging tests of patients with PELD, in which a marked involvement of the caudate is observed recurrently [1,58,59,60,61].

Some authors have shown that seipin could play an important role in the genesis of synaptic vesicles and their priming machinery necessary for the release of neurotransmitters. Thus, in cultured cortical neurons, the knockout *BSCL2* gene results in an impairment in the excitatory postsynaptic currents, while the inhibitory postsynaptic currents were normal [12]. The same authors [62] demonstrated that the N88S variant, responsible for seipin-related motor neuron diseases of the upper and/or lower motor neuron, could affect synaptic transmission by altering the docking and priming of synaptic vesicles.

Studies in seipin knockout mice have shown a particular action of this protein in the CNS that seems to be mediated by the peroxisome proliferator-activated receptor gamma (*PPAR***γ**) and that influences synaptic transmission mainly through alpha-amino-3-hydroxy-5-methyl-4-isoxazole propionic acid receptors, also promoting neuronal differentiation and modulating the behavior and motor skills of these animals [63,64,65]. In agreement with these findings, our group has shown [3], both in ex vivo studies of human brains and in neuronal cultures, that there is a relationship between the expression of *BSCL2* and that of *PPARG*.

On the other hand, our studies have found a robust relationship between the expression of *BSCL2* and different genes related to peroxisomes (*CAT*, *PEX1*, *PEX11G*, *PEX16*, *SOD1*), both in human brain samples and in SH-SY5Y cells differentiated to neurons in which *BSCL2* was overexpressed [3]. This is consistent with the recent finding of the close relationship between seipin and *PEX30* in the biogenesis of LD [28]. Finally, via confocal microscopy, we found that in hypothalamus samples from a patient with PELD, the expression of seipin was greatly reduced and that of *PEX16*, a protein required for peroxisome membrane biogenesis, was absent. Taken together, these findings are highly suggestive of a possible role for seipin in the proper functioning of peroxisomes, which, as discussed above, is critical in defending against oxidative stress in the brain.

## 4. Seipin-Associated Diseases: The Seipinopathies

Classically, seipinopathies refer only to the upper and/or lower motor neuron diseases due to variants in heterozygosity in the *BSCL2* gene [66], considering that CGL2, caused by pathogenic variants in homozygosity or compound heterozygosity in this gene, is not a seipinopathy. This does not seem to make much sense and, in the same way that the different conditions related to variants in the *LMNA* gene are called laminopathies [67], we understand that we must call all of those disorders caused by pathogenic variants in *BSCL2* “seipinopathies”.

As has been mentioned previously, certain variants in *BSCL2* can cause diseases in either simple heterozygosity, compound heterozygosity, or homozygosity. To date, simple heterozygous variants in *BSCL2* have been reported that give rise to upper and/or lower motor neuron diseases [68]. Recently, our group identified a variant in heterozygosity, arising from presumed parental germline mosaicism, leading to a picture of progressive epileptic encephalopathy in two Spanish children (vide infra) [69]. Recessive disorders related to *BSCL2* give rise to CGL2 or Berardinelli–Seip syndrome type 2 [2] and Celia’s encephalopathy [1], which could be considered a variant of CGL2.

### 4.1. Upper and/or Lower Motor Neuron Diseases

Certain missense variants in *BSCL2* that affect amino acids located in the N-glycation site situated in the luminal loop of the protein, p.(N88S), p.(S90L) and p.(S90W) in the *BSCL2*-205/207/210 transcripts (which affects the highly-conserved residues of the sequence consensus NXS/T at positions 88–90) give rise to upper and/or lower motor neuron diseases [68,70,71]. Later, the variant p.(R96H) was also associated with these conditions [72]. These neurological disorders are clinically heterogeneous, including Silver syndrome or spastic paraplegia 17 (SPG17; MIM: # 270685), variants of Charcot–Marie–Tooth disease type 2, and distal hereditary motor neuropathy type 5C (dHMN5C, MIM: # 619112) [68]. In the case of dHMN5C, the clinical picture may present as weakness and muscle wasting, predominantly in the hands, beginning in the fourth decade of life. The involvement can be symmetric or asymmetric, and muscle loss usually affects the thenar eminence and the interosseous muscles of the hands. Loss of sensation is rare, generally vibratory, but foot deformities are common. There is a decrease in motor nerve conduction velocities, with the sensory conduction being normal. There may be gait disturbances, which suggests the involvement of the pyramidal pathway [73]. Silver syndrome (MIM: #270685) is characterized by amyotrophy, weakness of the small muscles of the hand and mild to severe spasticity of the lower extremities, also indicating the involvement of the upper motor neurons. The heterogeneity of these disorders includes clinically asymptomatic individuals but with electromyographic abnormalities [68].

Ito et al. [74] demonstrated that these variants in *BSCL2* gave rise to aberrant forms of seipin that prevented the correct glycation of seipin, leading to an incorrect folding in the ER. This misfolding activated the unfolded protein response (UPR), including upregulation of pro-apoptotic transcription factor, CHOP, and cell death through ER stress. These same authors demonstrated that seipin is expressed in spinal cord motor neurons, which are the main cells affected in motor neuron diseases related to this protein.

### 4.2. Congenital Generalized Lipodystrophy type 2 (CGL2)

CGL2 is a recessive disorder characterized by a generalized absence of adipose tissue that is evident from birth or during the first year of life. These children have a triangular face, without the characteristic Bichat (buccal) fat pad. As a consequence of the lack of adipose tissue, the muscles are well-defined, acquiring a Herculean appearance over the years. The absence of subcutaneous fat explains the marked phlebomegaly in the upper and lower extremities. These children have early hyperinsulinemia, hypoleptinemia, hypoadiponectinemia, and hypertriglyceridemia. Over the years, and probably as a consequence of hyperinsulinemia, patients present acromegaloid features [75]. The ectopic deposition of fat in the liver leads to hepatic steatosis, which explains the large hepatomegaly, which can progress to cirrhosis. Due to their severe insulin resistance, these children develop acanthosis nigricans as early as the first few years of life, which worsens as they age. In addition, as a consequence of insulin resistance, these patients often develop diabetes mellitus, generally during the second decade of life, which is very difficult to control, generally requiring very high doses of insulin [76]. Unlike other subtypes of CGL, these patients frequently (80%) have a mild–moderate degree of intellectual disability [77]. Without treatment with recombinant human leptin (metreleptin) [78], the life expectancy of these patients is reduced. The most common causes of death are liver cirrhosis, infections, and complications of diabetes [79,80].

Most cases of CGL2 are due to homozygous or compound heterozygous variants which more commonly give rise to premature stop codons or frameshift, assuming a loss of seipin function.

### 4.3. Celia’s Encephalopathy

#### 4.3.1. Clinical Course of the Classic Form

**Figure 1:**
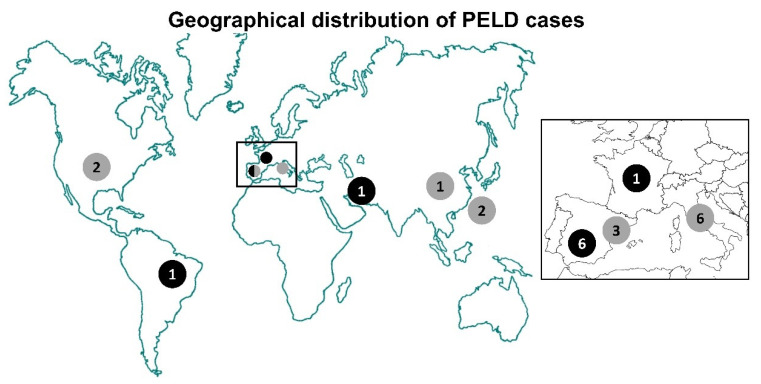
Worldwide distribution of *BSCL2* progressive encephalopathy with or without lipodystrophy. Black circles: Homozygous or compound heterozygous patients for c.985C>T variant; gray circles: Patients with other variants in *BSCL2*. The numbers inside the circles indicate the number of cases per country referred to in Table 1: Spain: Cases #1 to #6, #10, #22, and #23; Italy: Cases #12 to #14 and #19 to #21; USA: Cases #11 and #18; Taiwan: Cases #16 and #17; Brazil: Case #8; Iran: Case #7; China: Case #15; France: Case #9. See text for a more extended clinical description.

Celia’s encephalopathy is a fatal pediatric neurodegenerative condition due to the c.985C >T variant in *BSCL2* in exon 7, either in homozygosity or compound heterozygosity. Theoretically, this variant would lead to a premature stop codon p.(R329*). There are 9 known cases to date; 6 have died before the age of 9 years, (6–8 years), one died at the age of 28 years [59], one unpublished case is currently age 5 years, and one girl is currently 11 years of age (see below) (Figure 1). The cause of death was status epilepticus or pneumonia secondary to a progressive deterioration due to neurodegeneration.

These children are born apparently healthy, although they often have problems with sucking and weight loss in the first month. In our experience [1], homozygous patients did not show the characteristic features of generalized lipodystrophy, although they did have hypertriglyceridemia and hepatomegaly, which was resolved after a few months with a low-fat diet. Acanthosis nigricans or carbohydrate metabolism impairment have not been observed in any of the cases reported to date. In contrast, in compound heterozygotes, CGL2 traits were evident from the first months. However, other authors have reported at least one patient homozygous for c.985C >T with generalized lipodystrophy [81], and another Brazilian patient also homozygous for this variant who presented generalized lipoatrophy (unpublished data).

From the neurological point of view [1], these patients present a certain delay in the acquisition of developmental milestones after birth, with speech being particularly affected. In fact, most of these children can barely learn a few simple phrases. However, up to approximately three years of age, patients progress, although the acquisition of psychomotor skills is slower than those of their peers (they can walk, run, manipulate objects and understand simple commands, although they do not control sphincters). From the age of 3, a neurodevelopment regression is revealed, with a progressive loss of the skills acquired. Over the years, the neurodegenerative picture becomes more evident, with gait disturbance, frequent falls, loss of speech and understanding, the appearance of spasticity and the onset of epileptic signs in the form of initially subtle myoclonic seizures (blinking, drooling). As the disease progresses, they usually present with tonic-clonic seizures and other forms of epilepsy refractory to any type of anticonvulsant treatment, loss of head support, and spastic paraparesis. Finally, the patients present a complete disconnection from the environment, and difficulties in swallowing, with frequent choking, which generally leads to a deterioration of their nutritional status, forcing the placement of a percutaneous endoscopic gastrostomy (PEG) for artificial enteral feeding. As mentioned, death eventually occurs due to the deterioration of the patient’s general condition due to respiratory sepsis as a consequence of a poor nutritional status associated with bronchial aspiration or due to a status epilepticus which is not reversible with anticonvulsants or other therapies (ketogenic diet, adrenocorticotropic hormone treatment).

The most striking finding in the post-mortem studies performed on two of our patients revealed an intense atrophy of the caudate nuclei. In both cases, the caudate–putamen nuclei showed profound neuronal loss and astrogliosis, which was more intense in the dorsal and medial areas [1].

Strikingly, Poisson et al. [58] reported the case of a young woman, compound heterozygous for the variant c.985C>T and another variant in the *BSCL2* gene (c.1004A>C), who presented a different neurological clinical picture. She was diagnosed with regressive autism spectrum disorder, and developed an atypical Parkinsonism in adulthood. The neurological signs and symptoms began at 3 years of age with a slight behavioral disorder, which progressed to more invasive rituals, social withdrawal, attention problems, and sleep disorders. In a few months, the patient lost communication and language skills, and at 6 years of age she presented motor stereotypies, poor language, trichotillomania, and hypertonia of the lower limbs. At 16 years of age, her neurological deterioration was obvious, with bilateral dystonic hypertonia, and extrapyramidal and pyramidal signs. At the age of 21, she presented with camptocormia, at 23, falls and dysphagia appeared, and she was diagnosed with a marked frontal lobe syndrome. Brain magnetic resonance imaging (MRI) showed atrophy of the caudate nuclei. The patient died at 28 years of age.

This case is especially intriguing since it departs from the canonical clinical course of patients with the c.985C>T variant in compound heterozygosity, both in its neurological aspect (late onset of neurodegeneration, absence of obvious epileptic seizures, Parkinsonism) and in the absence of lipodystrophy, which is always present in compound heterozygotes. The second variant, c.1004A>C, also leads to skipping of exon 7, although the degree of expression of the aberrant transcript has not been established. It could be speculated that this case would be similar to the classic homozygous forms of PELD, but that the accumulation rate of the short isoform of seipin would be lower, which could justify the slower neurological involution.

#### 4.3.2. Molecular Bases and Pathogenetic Mechanisms

Theoretically, the c.985C>T variant, located in exon 7 of the *BSCL2*-203 transcript, should give rise to a premature stop codon, p.(Arg329*). However, studies carried out by our group [1] have shown that the transition from cytosine to thymine at this position gives rise to a consensus sequence CTRAY (CCGAC >> CTGAC) for the branch site, which leads to complete intronization of exon 7 [1].

As a result of exon 7 skipping, an aberrant seipin is transcribed in which the second transmembrane domain of seipin disappears, so that from the first transmembrane domain the rest of the protein remains housed in the ER lumen. This seipin could correspond to the short isoform, 287 amino acids, or to a new seipin (Celia seipin) of 287 + 64 amino acids (by including the transcription of exon 1), as we have been able to verify through qPCR studies in brain samples of the index case (unpublished data). These qPCR studies showed that in the brain of the index case this transcript without exon 7 was expressed up to 21% compared to 0.3% in brains of healthy individuals. Also interesting was the fact that the expression of this transcript was also very high in non-nervous tissues of the index case [1]. Whether this high expression in the PELD patient could confer some kind of protection against lipodystrophy is a matter that has not yet been unveiled. On the other hand, in the primary preadipocytes of the index case, we were able to observe by electron microscopy a frank dilation of the ER cisterns, filled with proteinaceous material [1]. Through overexpression studies in different cell models, we were able to demonstrate that this new form of seipin induced ER stress, in line with the observations by electron microscopy previously discussed [1,34]. On the other hand, in brain samples from the index case, we observed the presence of ubiquitin-positive intranuclear inclusions in the neurons of the index case [1]. Later [34], we were able to demonstrate by immunohistochemistry that these inclusions correspond to seipin macroaggregates. At first, it may be striking to find seipin, an ER protein, inside the nucleus. In our first studies in HeLa cells, in which we overexpressed both whole *BSCL2* and *BSCL2* without exon 7, we verified, through subcellular fractionation analysis, the presence of seipin in the nucleus, and more markedly in cells that overexpressed Celia seipin [1,34]. Similarly, by immunofluorescence microscopy, we observed a more marked seipin ring around the nucleus. These inclusions were mostly single and round-shaped, although occasional multiple inclusions were also observed. All of this is consistent with recent findings that place seipin in the outer nuclear membrane in mammalian cells [32], and not in the INM as occurs in yeast [31]. Given that there is an increase in the formation of nuclear LD in stress situations [30], it could be hypothesized that the neuronal damage induced by ER stress in PELD could facilitate the passage of seipin molecules to the nucleoplasm forming aggregates, which could participate in its pathogenesis, in a similar way to Huntington’s disease [82].

We are therefore faced with a variant in *BSCL2* that leads to a toxic function gain, but in a recessive way, which is surprising. Theoretically, simple heterozygous carriers should present some type of neurological symptoms. However, this has been ruled out by studying all the parents of affected children [1]. In an attempt to find an explanation for this fact, our group carried out co-immunoprecipitation studies in cells in which the wild-type (wt) and the mutated transcript were overexpressed in different proportions, comparing the interaction between wt seipin and Celia seipin, demonstrating that both proteins interact. In addition, by density gradient fractionation, we observed a change in the buoyancy pattern of Celia seipin at a 10:1 ratio of Myc-wt/Myc-Celia seipin. Specifically, at a 10:1 ratio Celia seipin migrates at a lighter fraction than when alone, mimicking the behavior of wt seipin. These results suggest that, in simple heterozygotes, wt seipin could be sequestering Celia seipin, preventing ER stress and therefore the response to misfolded proteins. Due to the absence of wt seipin, in compound heterozygous patients, this proposed phenotype rescue would not be possible [34]. Rescue of protein aggregates by a well-folded wt conformer is a very interesting molecular phenomenon, which to the best of our knowledge has never described before in the realm of neurodegenerative diseases caused by protein misfolding and/or aggregation. It could be said that the wt seipin acts as an “anti-prionoid” in this situation [83].

On the other hand, the fact of having found a strong correlation between the expression of *BSCL2* and different genes of the peroxisomes, that the overexpression of *BSCL2* in neuronal cultures increased their expression, and that in confocal microscopy studies of hypothalamus samples of the index case the presence of PEX16 was not observed, led us to hypothesize that, beyond the proposed mechanisms of neuronal death in PELD, a poor functioning of the peroxisomes could be contributing to the neurodegenerative phenotype [3,39,40].

In summary, in Celia’s encephalopathy, the excessive synthesis of an aberrant seipin isoform, mainly in the CNS, leads to ER stress and the formation of intranuclear aggregates, which would lead to neuronal death. Why lipoatrophy does not manifest itself in some homozygote patients, at least not in a serious form, is something that has yet to be elucidated.

### 4.4. Other Variants in BSCL2

All variants are referred to transcript NM_001122955.3 of *BSCL2* (Table 1).

#### 4.4.1. Variant c.974dupG

This variant was first reported in 2003 by Agarwal et al. [77] as c.1126insG, in heterozygosity, in a 9-year-old girl with CGL2 and an intellectual disability (Table 1, patient 18). Years later, in 2009, Wu et al. [84] reported the case of a 28-year-old male, compound heterozygote, c.[974dupG];[757G>T], in the original c.[783insG];[565G>T] (Table 1, patient 16). The patient had CGL2 (with muscular hypertrophy, acromegaloid features, acanthosis nigricans, diabetes, hypertriglyceridemia, and hepatic steatosis) and neurological symptoms (mild intellectual disability, gait disorders, axial dystonia, and behavioral disturbances), but no seizures or encephalopathy. Imaging tests were normal. One year later, Huang et al. [85] reported the case of a 1.8-year-old boy with CGL2 (with well-defined musculature, severe hypertriglyceridemia and hepatic steatosis) due to the homozygous c.974dupG variant (c.783insG in the original) without neurological involvement, although there was no evidence of subsequent follow-up (Table 1, patient 17). Neurological imaging tests were not performed. In 2016, Opri et al. [59] reported three cases of CGL2 associated with progressive epileptic encephalopathy (Table 1, patients 12–14). They were two girls homozygous for c.974dupG (in the original: c.782_783dupG), and a compound heterozygous boy (in the original: c:[782_783dupG];[828_829delAA]), who died between the ages of 7 and 11. The three patients presented CGL2, with well-defined musculature, hypertriglyceridemia, acanthosis nigricans, hepatic steatosis, and hypertrophic cardiomyopathy, in at least two of the cases. These patients presented a neurological picture superimposable to Celia’s encephalopathy. The neurological symptoms began at 4–5 years of age as an absence seizure, myoclonus, and ataxic gait. Neurological involution was rapid with the appearance of pyramidal signs, loss of language, severe intellectual impairment with dystonic tetraplegia, and continuous myoclonus. The epilepsy ranged from absence seizure to myoclonic-atonic seizures. Neuroimaging tests showed a particular involvement of the head of the caudate and lenticular nuclei, associated with a hypertensive signal both in these structures and in the periventricular white matter.

In 2018, our group reported two cases of CGL2 associated with the c.974dupG variant, one in homozygosity and the other in compound heterozygosity (c.[974dupG];[1015C>T]) (Table 1, patients 10 and 11) [60]. The first case was a girl with generalized lipodystrophy, hypertriglyceridemia, and hepatomegaly, although the latter two disorders resolved in a few months with low-fat diet. However, she did not present acanthosis nigricans, phlebomegaly, or muscle hypertrophy. From the age of two, the delay in language acquisition was evident, although the rest of the nervous system functions were reasonably normal, including her relationship with relatives and peers. However, the girl acquired relatively complex language. From the age of 5, she presented a regression in language and psychomotor skills, and a year later, she could hardly speak, also presenting an alteration in gait with frequent falls. At that age, she had stereotypical movements with orolingual dystonia. The neurological deterioration was progressive in the following months with dysphagia, loss of the ability to walk, pyramidal signs, spasticity, hyperreflexia, and severe cognitive deterioration. From the age of 9, she presented prolonged tonic-clonic epileptic seizures and died at the age of 9 years and 9 months. Neuroimaging tests revealed a marked involvement of the caudate nuclei and a marked hypometabolism in the parietal and occipital lobes, similar to what was observed in one of our patients with the classic form of PELD (patient 2 in Table 1) [1,60]. On the other hand, the other patient, aged 2, in compound heterozygosity, presented a classic CGL2, with hypertriglyceridemia and hepatomegaly, and a very mild language impairment.

Finally, in 2019, Zhang et al. [86] reported the case of a 17-year-old man with CGL2 due to the homozygous c.974dupG variant (c.782dupG in the original). This patient presented intellectual disability and severe epileptic signs and symptoms from the age of three, refractory to different anticonvulsant treatments (Table 1, patient 15).

In view of these cases, mainly those reported by Opri et al. [59] and one of our patients, we considered the possibility that the mechanism responsible for the neurodegenerative picture could be the same as that found in Celia’s encephalopathy. The molecular studies carried out in our laboratory confirmed our hypothesis. Thus, the c.974dupG variant, located in exon 7 of the *BSCL2* gene, also gave rise to skipping of this exon, in exactly the same way as in the classic form of PELD [1,60]. Our experiments demonstrated that the c.974dupG variant alters certain splicing ancillary sequences called exonic splicing enhancers (ESE), creating a new ESE. Our qPCR studies for the transcript that encodes Celia seipin performed in primary fibroblasts from one of our patients showed a significant increase in the expression of the said transcript, albeit in a lower proportion than that observed in the samples of the index case of PELD [60].

#### 4.4.2. Variant c.1048C>T

In 2020, Ferranti et al. [61] reported the case of two sisters, aged 11 and 18, who presented a picture of progressive epileptic encephalopathy with movement disorders and CGL2, although the clinical description of the lipodystrophic phenotype was poor (Table 1, patients 19 and 20). Both sisters carried the c.1048C>T (exon 8) biallelic variant in the *BSCL2* gene, as their parents were asymptomatic carriers. This variant would lead to the appearance of a premature stop codon, p.(Arg350*). The two patients manifested an early delay in language acquisition. From the age of 5, they presented generalized epileptic seizures, with a neurological worsening over the years, including myoclonic seizures, and dyskinetic, dystonic, and choreoathetotic movements. The epileptic disorders were resistant to different combinations of anticonvulsant drugs. In late childhood, these girls presented psychomotor agitation, progressive language impairment, and signs of bulbar involvement. At the age of 10, both sisters lost their gait, and at 15 the elder became aphasic, dying at 18 as a result of the deterioration of her general condition. MRI revealed supra and infratentorial atrophy, and a shrinkage of the caudate and putamen nuclei.

It is clear that these conditions are superimposable with Celia’s encephalopathy, although the life expectancy was somewhat longer. We have analyzed this variant in our laboratory, and it is striking that, although the c.1048C>T variant would cause a premature stop codon (R[CGA] > *[TGA]) in long seipin (462 aa), as well as in the NM_032667.6 transcript, which encodes for the 398 amino acid seipin, (c.856C>T, p.(Arg286*), R[CGA] > *[TGA]), this does not occur for the NM_001130702.2 transcript, which encodes for short seipin (287 amino acids) and which is directly related to the pathogenesis of Celia’s encephalopathy. In this transcript, the c.714C>T variant would not encode for a premature stop codon (p.(Asn238=), N[AAC] > N[AAT]) [87]. The stop codon that appears in both transcripts NM_001122955.3 (which encodes for seipin of 462 amino acids) and NM_032667.6 (which encodes for seipin of 398 amino acids) fulfills the rule of 5–55 nucleotides [88], and therefore it could be assumed that these transcripts would be degraded by the nonsense mediated decay (NMD) pathway, making it possible to hypothesize an overexpression of an aberrant longer isoform of Celia seipin, in line with the proposed pathogenetic mechanisms, although these expression studies have not been carried out.

Although these authors did not conduct gene expression studies, taking into account both the neurological and lipodystrophic clinical pictures, and the results of neuroimaging tests, also superimposable to those found in PELD, it would not be unreasonable to hypothesize that variant c. 1048C>T would lead to a loss of function of the long and intermediate seipin isoforms and to an overexpression of the short isoform, as occurs in PELD, although this is something that must be verified experimentally.

#### 4.4.3. Variant c.1076dupC

In 2019, Serino et al. [89] published the case of a child with CGL2, myoclonic epilepsy, and progressive neurological deterioration due to the variant c.1076dupC (exon 9) in the *BSCL2* gene (Table 1, patient 21), another condition that could be considered a new form of Celia’s encephalopathy [90]. This variant would give rise to a premature stop codon in the transcript NM_001122955.3, p.(Glu360*), [CCT (Pro) GAA (Glu) >> CCC (Pro) TGA (*)]. For the NM_032667.6 transcript, the variant notation would be c.884dupC, which would also lead to a premature stop codon, p.(Glu295*). However, in the NM_001130702.2 transcript, which encodes the short isoform, the insertion of a cytosine (c.742dupC) does not give rise to a premature stop codon, but rather to a frameshift which causes an elongation of 83 amino acids at the end intraluminal carboxy-terminal domain, for which a 370-amino-acid-long seipin would be translated, and not the 287 amino acids of the short canonical isoform of seipin, p.Leu228Glu*fs*TER103. Although the premature stop codons predicted in the long and intermediate transcripts do not comply with the 50–55 nucleotide rule as an NMD mechanism [88], it might be possible that this occurs via other mechanisms not related to the presence of exon junction complex [91]. In this sense, it has been suggested that mRNA degradation due to the presence of a premature stop codon could influence a higher expression of other transcripts [92].

#### 4.4.4. Variant c.566T>A

In 2019 our group [69] reported the case of two brothers with a picture of epileptic encephalopathy and neurodevelopmental involvement, albeit with no evidence of CGL2 (Table 1, patients 22 and 23). Using whole-exome sequencing in a family quartet, we identified the missense variant c.566T>A in heterozygosity in exon 4 of the *BSCL2* gene, which would lead to the change p.(Met189Lys) in the luminal loop of the protein as potentially responsible for the neurological picture. It would be a de novo variant due to germline mosaicism. The expression studies in fibroblasts from one of the patients did not find differences in the expression of the different *BSCL2* transcripts. The neurological signs and symptoms began early in both siblings, with a delay in the acquisition of developmental milestones and frequent febrile seizures. The older of the siblings presented myoclonus and episodes of absence from the age of two, as well as atonic seizures. From the age of 4, generalized tonic seizures were very frequent with a poor response to different anticonvulsant treatments, including the ketogenic diet. Currently, at the age of 11, the child presents an intellectual deficit, an autism spectrum disorder, ataxic gait, and a significant impairment of language. The patient’s younger sister began to present neurological symptoms at three months of age, with asymmetric tonic seizures at 8 months. Death occurred at the age of 10 months due to refractory status epilepticus. Imaging tests were unremarkable for both siblings. Neuropathological studies of the deceased patient revealed microglial activation and astrogliosis with the formation of microglial nodules in the basal ganglia, although no nuclear inclusions were observed.

This is an especially relevant case since it neurologically mimics Celia’s encephalopathy, although the variant in *BSCL2* is heterozygous and does not affect short seipin. On the other hand, this variant gives rise to an amino acid change in the intraluminal domain, as occurs in diseases of motor neurons associated with *BSCL2*. The mechanism responsible remains to be clarified.

## 5. Treatment

Celia’s encephalopathy has no cure and leads to early death in most patients, generally before the age of ten, with a few exceptions. The cause of death is directly related to the neurodegenerative process and/or uncontrollable epilepsy.

The therapeutic approach to these patients has two aspects: 1. The control of metabolic complications in those cases in which generalized lipodystrophy is present, and 2. the control of epileptic seizures and early psychomotor care. This approach must be multidisciplinary, involving both neuropediatricians and endocrinologists with expertise in lipodystrophies.

In the first aspect, in general, diets low in fat and low in simple carbohydrates, with preference for high-fiber complex carbohydrates [93], associated or not with supplements with omega-3 fatty acids and/or medium chain triglycerides, seem to quickly and efficiently improve hypertriglyceridemia and hepatic steatosis. It should be emphasized that these children should not be overfed. Due in many cases to the marked lipoatrophy they suffer, they can give the impression that they are malnourished, which can lead to an increase in caloric intake, thus leading to a worsening of metabolic complications. However, sufficient caloric intake must be guaranteed for proper development, focusing more on growth than on body mass index. In the event that the ability to swallow is compromised or absent, the placement of a PEG for artificial enteral feeding should be chosen.

The treatment of the epilepsy crisis is challenging as these patients’ seizures are often refractory to any type of anti-seizure drug. Adrenocorticotropic hormone treatment has been attempted without much success and glycemic control must be closely monitored. The ketogenic diet has also been tried with no apparent results. The implantation of a vagus nerve stimulator could temporarily improve the frequency of seizures [89].

Since 2012, our group has been treating a girl with Celia’s encephalopathy in its classic form (Table 1, patient 2) with recombinant human leptin and omega-3 fatty acid supplementation [94]. The rationale for this therapeutic approach is based in part on the fact that these patients are severely hypoleptinemic. Leptin is known to have a beneficial effect on CNS functions [95,96] and on some neurodegenerative disorders [97,98]. In addition, leptin regulates the synapse morphology of hippocampal neurons [99] and modulates the development of oligodendroglial cells [100], which can contribute to structural changes in gray matter. Leptin may also help prevent neuronal death in neurodegenerative disorders [96]. On the other hand, omega-3 fatty acids, and in particular docosahexaenoic acid (DHA), modulate neurogenesis, synaptogenesis, and neurite growth; refine synaptic connectivity; control the release of neurotransmitters; and play a role in memory consolidation processes [101,102,103,104]. Therefore, dietary modifications and supplementation could have neuroprotective and therapeutic effects [105]. On the other hand, and in a more specific way, our group demonstrated in vitro the beneficial effect of treatment with leptin and polyunsaturated fatty acids by reducing the expression of the aberrant transcript of *BSCL2* [94,106]. This therapeutic approach managed not only to slow down neurological involution and the appearance of seizures, but also to improve glucose consumption in serial positron emission tomography studies performed over several years of follow-up [94].

In view of the results of our studies and those of other authors on the relationship between seipin, peroxisomes, and protection against oxidative stress [3,107,108], in 2018, we added to the treatment (which also included valproic acid and clonazepam) two peroxisome proliferator-activated receptors agonists; a PPARα agonist, fenofibrate, and a PPARγ agonist, pioglitazone. Both drugs were well tolerated. Different studies have shown that the activation of peroxisome proliferator-activated receptors has anti-inflammatory effects that were beneficial in several mouse models of neurodegenerative diseases including ALS [109,110,111], tauopathy [112], Huntington’s disease [113], experimental autoimmune encephalomyelitis, [114] and Parkinson’s disease [115]. Although no experimental data are available on the effect of both drugs on the pathogenetic mechanisms of Celia’s encephalopathy, to date and at the age of 11 years and 6 months, the patient, although severely affected from a neurological point of view, can walk with support, understand some simple commands, is able to partially swallow, and does not present epileptic seizures with the exception of some palpebral myoclonus.

Along the same lines, another of the patients with the c.974dupG variant has been receiving metreleptin and fish oil since the age of 6 months and, at the time of writing (Table 1, patient 11), at 4.5 years of age, she does not present neurological symptoms, attends preschool and receives speech therapy but otherwise seems to have normal cognition (Dr. Melissa Crocker, personal communication). In addition, recently, Pedicelli et al. [90] have reported that treatment with metreleptin for one year reduced the number of epileptic seizures in an 11-year-old boy with the homozygous c.1076dupC variant [89].

## 6. Conclusions

Seipinopathies are a heterogeneous group of diseases that affect adipose tissue and the nervous system. Heterogeneity is both phenotypic and allelic, and can present as dominant or recessive disorders. From a clinical point of view, these disorders can manifest as more or less disabling neuropathies, generalized lipodystrophy associated with intellectual disability, progressive epilepsy, or lethal neurodegenerative conditions such as Celia’s encephalopathy. Although in the case of type 2 CGL it can be assumed that the pathogenetic mechanism is a consequence of a loss of seipin function, in neurological conditions, both dominant and recessive, there is solid evidence to conclude that neurological damage is related to a gain of toxic function.

In the particular case of PELD, it can be concluded that, regardless of the variant in *BSCL2* responsible for the disease, overexpression of the short isoform of seipin is at the root of the neurological damage, at least in the majority of the reported cases. The role that seipin accumulation may play in the nucleus and ER, or a hypothetical malfunction of peroxisomes in the CNS, is something that deserves further study in the future.

Although there is no curative treatment for this condition, the few published therapeutic approaches suggest that recombinant human leptin and omega-3 fatty acids could help slow down the process and control seizures in these children.

Finally, the considerable similarities of the long isoform of seipin in primates, but especially in the great apes, the differences in the expression of *BSCL2* between humans and rodents in the brain, and the reduction of its expression in the human brain during aging [3] raise attractive hypotheses regarding the role of seipin in the encephalization process and in the etiopathogenesis of other, more common, neurodegenerative diseases.

## Figures and Tables

**Table 1:** Progressive encephalopathy with or without lipodystrophy (PELD) *BSCL2* variants referred to transcript NM_001122955 3 geographical distribution of PELD cases is depicted in Figure 1.

Case	BSCL2 Variant	BSCL2 Reported Variant	Genotype	Sex	Reported Age	GL	Encephalopathy	Seizures	Other Neurological Symptoms	Other Non-Neurological Symptoms	Treatment	Deceased (age, years)	Ref.
1	985C>T	985C>T	Homozygote	F	D	N	Y	Generalized tonic-clonic	Poor motor coordination, ataxic gait, generalized fine tremor, dystonia, sleep disturbances, severe spasticity, tetraparesis, pyramidal and extrapyramidal signs, loss of motor skills, social, language and cognitive development	Hypertriglyceridemia, hepatic steatosis	Antiepileptic	Y (8)	[1]
2	985C>T;507_511del	985C>T;507_511del	Compound heterozygote	F	11	Y	Y	Progressive myoclonic epilepsy	Gait ataxia, intellectual disability, dystonia, difficulty swallowing, loss of language	Hypertriglyceridemia, hepatic steatosis	Anti-epileptic, metreleptin, n-3 FA, fenofibrate, pioglitazone	N	[1]
3	985C>T;538G>T	985C>T;538G>T	Compound heterozygote	M	D	Y	Y	Progressive myoclonic epilepsy	Cognitive impairment, pyramidal signs, language loss, dystonia, difficulty swallowing	N.R.	Antiepileptic	Y (8)	[1]
4	985C>T;507_511del	985C>T;507_511del	Compound heterozygote	M	D	Y	Y	Progressive myoclonic epilepsy	Cognitive impairment, ataxic gait	N.R.	Antiepileptic	Y (7)	[1]
5	985C>T;507_511del	985C>T;507_511del	Compound heterozygote	M	D	Y	Y	Progressive myoclonic epilepsy	Cognitive impairment, ataxic gait	Hypertriglyceridemia, hepatic steatosis	Antiepileptic	Y (7)	[1]
6	985C>T	985C>T	Homozygote	F	D	N	Y	Progressive myoclonic epilepsy	Cognitive impairment, irritability, dysphagia, sleep disorder and pyramidal signs, ataxic gait	N.R.	Antiepileptic	Y (6)	[1]
7	985C>T	985C>T	Homozygote	M	D	Y	Y	Myoclonic	Autism, repetitive and stereotypic hand movements, repetitive upward staring, ataxia, generalized hypertonia, severe global developmental delay	Inguinal hernia, generalized hypertrichosis	Antiepileptic	Y (8)	[81]
8	985C>T	Not published	Homozygote	M	5	Y	Y	Myoclonic	Gait ataxia, intellectual disability	Hypertriglyceridemia, hepatic steatosis	Antiepileptic	N	(not published)
9	985C>T;1004A>C	985C>T;1004A>C	Compound heterozygote	F	D	N	Y	N	Regressive autism spectrum disorder, atypical Parkinsonism, loss of communication and language skills, dystonic hypertonia, and extrapyramidal and pyramidal features, camptocormia, dysphagia marked frontal lobe syndrome	N.R.	None	Y (28)	[58]
10	974dupG	974dupG	Homozygote	F	D	Y	Y	Myoclonic	Language delay, myoclonus, dystonia, seizures, gait ataxia, abnormal behavior	Hypertriglyceridemia, hepatic steatosis	Antiepileptic	Y (9)	[60]
11	974dupG;1015C>T	974dupG;1015C>T	Compound heterozygote	F	2	Y	N	N	Language delay	Hypertriglyceridemia, hepatic steatosis, hyperinsulinemia	Metreleptin, n-3 FA	N	[60]
12	974dupG;1020_1021delAA	782_783dupG; 828_829delAA	Compound heterozygote	M	D	Y	Y	Absence seizures; eyelid myoclonus	Pyramidal signs, loss of language, dystonic tetraplegia	Hypertriglyceridemia, hypertransaminasemia, hepatic steatosis, hypertrophic cardiomyopathy	Antiepileptic	Y (9)	[59]
13	974dupG	782_783dupG	Homozygote	F	D	Y	Y	Absence seizures, slight eyelid myoclonus, myoclonic-atonic seizures	Cognitive decline, pyramidal signs, tetraparesis, intellectual disability	Hypertriglyceridemia, muscle hypertrophy, hepatic steatosis, cardiomegaly, acanthosis nigricans	Antiepileptic	Y (7)	[59]
14	974dupG	782_783dupG	Homozygote	F	D	Y	Y	Absence seizures, slight eyelid myoclonus, myoclonic-atonic seizures	Cognitive decline, pyramidal signs, tetraparesis, intellectual disability	Hypertriglyceridemia	Antiepileptic	Y (11)	[59]
15	974dupG	782dupG	Homozygote	M	17	Y	Y	Progressive myoclonic epilepsy	Generalized hypertonia	None	Antiepileptic	N	[86]
16	974dupG; 757G>T	783insG;565G>T	Compound heterozygote	M	28	Y	N	N	Gait disturbance, torticollis, abnormal posturing of the fingers, axial dystonia abnormal thigh abduction and foot dystonia, mild intellectual disability	Muscular hypertrophy, acromegaloid features, acanthosis nigricans, hypertriglyceridemia, hepatic steatosis	—	N	[84]
17	974dupG	783insG	Homozygote	M	1.8	Y	N	N	None	Prominent musculature, eruptive xanthomas, hepatic steatosis, hypertriglyceridemia, hyperinsulinemia.	Fenofibrate	N	[85]
18	974dupG	1126insG	Heterozygote	F	9	Y	N.R.	N.R.	Intellectual disability	Diabetes mellitus	N.R.	N.R.	[77]
19	1048C>T	1048C>T	Homozygote	F	15	Y	Y	Progressive myoclonic epilepsy	Dyskinetic, distonic, and choreoathetotic movements, psychomotor regression, language impairment, bulbar signs, loss of gait	Coarse facial features, synophrys, bulbous nasal tip, large ear pinnae, wide mouth, long fingers and toes, hypertrichosis	Antiepileptic	N	[61]
20	1048C>T	1048C>T	Homozygote	F	18	Y	Y	Progressive myoclonic epilepsy	Dyskinetic, distonic, and choreoathetotic movements, psychomotor regression, language impairment, bulbar signs, loss of gait, aphasia	Coarse facial features, synophrys, bulbous nasal tip, large ear pinnae, wide mouth, long fingers and toes, hypertrichosis, abnormal glucose tolerance	Antiepileptic	Y (18)	[61]
21	1076dupC	1076dupC	Homozygote	M	6	Y	Y	Progressive myoclonic epilepsy	Language impairment, severe hyperactivity, drop attacks, ataxic gate, psychomotor delay, ataxic gait	Hypertriglyceridemia, hypertransaminasemia, hepatic steatosis	Antiepileptic	N	[89]
22	566T>A	566T>A	Heterozygote	M	10	N	Y	Generalized tonic-clonic	Delays in social skills, eyelid myoclonus, absence, atonic seizures, moderate intellectual disability, autism spectrum disorder, ataxic gait	None	Antiepileptic	N	[69]
23	566T>A	566T>A	Heterozygote	F	0.9	N	Y	Asymmetric tonic seizures	Mild psychomotor delay	None	Antiepileptic	Y (0.9)	[69]

Y: Yes; N: No; D: Deceased; GL: Generalized lipodystrophy. N.R.: Not reported; n-3 FA: omega-3 fatty acids.

## Data Availability

No new data were created or analyzed in this study. Data sharing is not applicable to this article.
